# Sensing and Detection of Traffic Signs Using CNNs: An Assessment on Their Performance

**DOI:** 10.3390/s22228830

**Published:** 2022-11-15

**Authors:** Lorenzo Canese, Gian Carlo Cardarilli, Luca Di Nunzio, Rocco Fazzolari, Hamed Famil Ghadakchi, Marco Re, Sergio Spanò

**Affiliations:** Department of Electronic Engineering, University of Rome “Tor Vergata”, via del Politecnico 1, 00133 Rome, Italy

**Keywords:** traffic sign, convolutional neural network, deep learning, CNN, dataset

## Abstract

Traffic sign detection systems constitute a key component in trending real-world applications such as autonomous driving and driver safety and assistance. In recent years, many learning systems have been used to help detect traffic signs more accurately, such as ResNet, Vgg, Squeeznet, and DenseNet, but which of these systems can perform better than the others is debatable. They must be examined carefully and under the same conditions. To check the system under the same conditions, you must first have the same database structure. Moreover, the practice of training under the same number of epochs should be the same. Other points to consider are the language in which the coding operation was performed as well as the method of calling the training system, which should be the same. As a result, under these conditions, it can be said that the comparison between different education systems has been done under equal conditions, and the result of this analogy will be valid. In this article, traffic sign detection was done using AlexNet and XresNet 50 training methods, which had not been used until now. Then, with the implementation of ResNet 18, 34, and 50, DenseNet 121, 169, and 201, Vgg 16_bn and Vgg19_bn, AlexNet, SqueezeNet1_0, and SqueezeNet1_1 training methods under completely the same conditions. The results are compared with each other, and finally, the best ones for use in detecting traffic signs are introduced. The experimental results showed that, considering parameters train loss, valid loss, accuracy, error rate and Time, three types of CNN learning models Vgg 16_bn, Vgg19_bn and, AlexNet performed better for the intended purpose. As a result, these three types of learning models can be considered for further studies.

## 1. Introduction

Deep learning (DL), or deep structured learning (DSL), is part of machine learning (ML), which is based on artificial neural networks (ANN). There are three different types of learning that can be used: supervised, semi-supervised, and unsupervised [1,2,3]. Moreover, for each of the named types, a novel research field consists of their hardware acceleration [4,5,6]. This means that there is a need for improved and efficient machine learning systems.

The architecture of DL, which has been applied to different fields such as computer vision, drug design, image processing, board game programs, medical image processing, driver identification, etc., such as deep neural networks (DNN), deep reinforcement learning (DRL), convolutional neural networks (CNN), etc. [7,8,9,10,11,12].

With the ongoing development of deep learning, computer vision has made significant progress and is now applicable to online situations. One of the current subtasks of computer vision is object recognition. It is widely used in intelligent transportation applications such as motorized and non-motorized vehicle detection, pedestrian detection, and self-driving cars. This study, which focuses on traffic sign recognition, is critical for creating highly accurate maps for driverless automobiles. Traffic signs are widely scattered in real traffic situations, and weather conditions, such as fog, rain, and snow, can also affect recognition accuracy. In this case, it is undoubtedly fatal for drivers and pedestrians. Therefore, it is especially important to enhance traffic sign detection performance in a variety of complicated situations [13].

Researchers’ contributions to convolutional neural networks have helped computer vision advance quickly in recent years. Prior to CIFAR [9], only small, low-resolution datasets could be used to train neural networks for classification tasks. Then, Alex et al. [9] presented AlexNet to accomplish the classification task in sizable datasets such as ImageNet [14]. AlexNet consists of convolutional and fully connected layers. Following that, scientists suggested networks with more layers, such as VGG [15], which somewhat increased the accuracy of networks. At a certain depth, the gradient will, however, diminish or disappear. In order to ensure that deeper network layers can obtain no fewer features than shallower network layers, He et al. [16] proposed ResNet, which uses cross-layer connections to fuse the input with the output of the residual blocks. This effectively prevents the phenomenon of insignificance or even the disappearance of deeper features. By modifying the depth, width, and input image resolution of the network model, the majority of the earlier networks enhance network performance. In order to combine the three factors, Tan et al. [17] introduced EfficientNet, which first constructed an ideal benchmark and then modified the benchmark network based on various scaling factors.

In deep learning, a CNN (or ConvNet) is one of the ANNs, most commonly applied to analyzing visual imagery [18]. It is a regularization of multilayer perceptrons. It usually means fully connected networks. It means that the neurons in one layer are fully connected to all the neurons in the next layer.

In CNNs, to make complex patterns, they make simpler patterns by assembling them, so in terms of connectivity and complexity, CNNs are on the lower extreme. Another important advantage in comparison with other image processing algorithms is preprocessing. This means that the filters learn to optimize through automated learning. This independence from prior knowledge and human intervention in feature extraction is a major advantage.

A very good example of DL is driver identification. Simply, according to claims, the Intelligent Transportation System (ITS) will transform travel by enhancing people’s safety, security, and comfort. Although some vehicles are automated, there are still serious security concerns that need careful investigation and cutting-edge solutions. The attacker can steal the vehicle thanks to ITS’s security flaws. Therefore, in order to create a safe and secure system and safeguard the vehicles from theft, the identification of drivers is necessary. A driver can be recognized in two ways: (1) by face recognition of the driver, and (2) based on driving behavior. Face recognition involves processing 2-D images for images and learning the features, both of which need a lot of processing power.

Traffic sign recognition has two varying approaches that have been utilized by researchers in recent years. One-stage networks, such as R-FCN [19], Faster R-CNN [20], Mask R-CNN [21], etc., and two-stage networks, such as YOLO [22] and SSD [23], improve the speed and accuracy of detection and classification. Both strategies have pros and cons and can be used differently. One-stage detectors, such as YOLO, are great for identifying small objects, but they struggle with large numbers of classes or classes that are identical to one another. When the traffic sign is too far away, blurry, or partially covered by other objects, the accuracy of the two-stage detectors drops dramatically [24,25,26].

## 2. Structure of Different CNN Models

**Figure 1:**
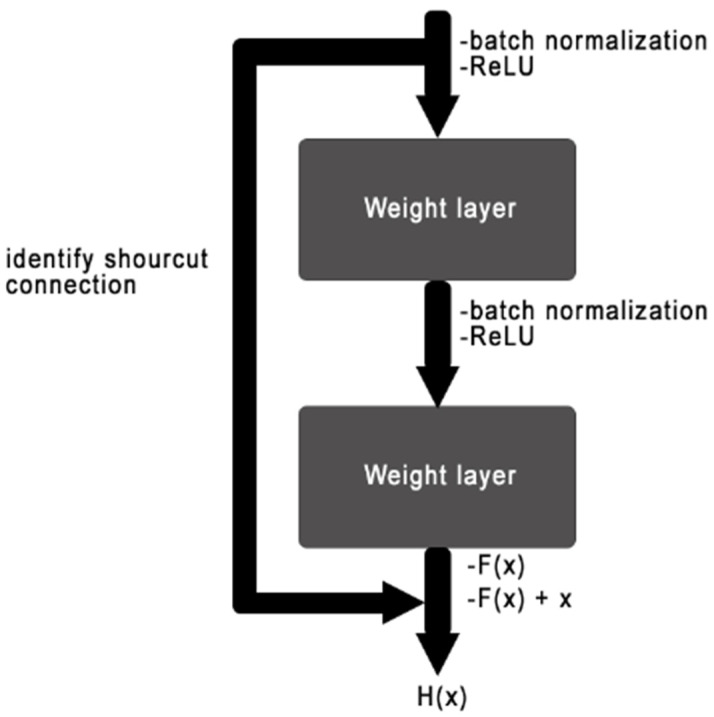
A residual block, as used in ResNets.

The most important models of CNNs are ResNet, XRexNet, DenseNet, VGG, SqueezeNet, and AlexNet. ResNets are one of the powerful systems for deep learning that were presented by Kaiming He et al. [16]. He has achieved the training system with good performance. As a result, ResNet became one of the most widely used systems for computer vision tasks. The important idea of ResNet is to introduce residual blocks that include an “identity shortcut connection” that skips one or more layers, as shown in Figure 1. 

This residual block changes the goal of the stacked layers from fitting the ideal weights and biases H(x) to fitting the output of the ResBlock, H(x) = F(x) + x [17].

The CNNs begin with an input, which is followed by four different stages, and each stage has similar patterns.

**Figure 2:**
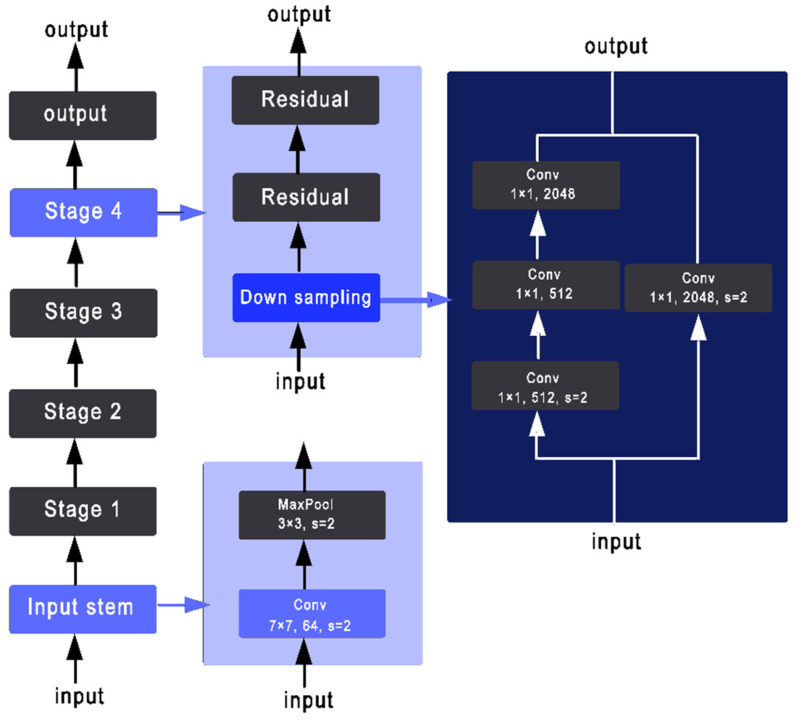
ResNet50 architecture.

With increasing the size of the ResNet, the accuracy of the prediction and also the time of the training will increase. The architecture of a ResNet50 is shown in Figure 2.

**Figure 3:**
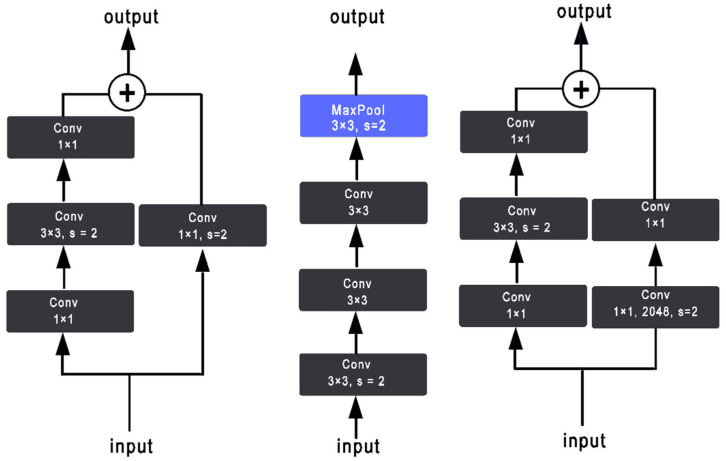
Three ResNet methods. ResNet-B modifies the downsampling block of Resnet. ResNet-C further modifies the input stem. On top of that, ResNet-D again modifies the downsampling block.

**Figure 4:**
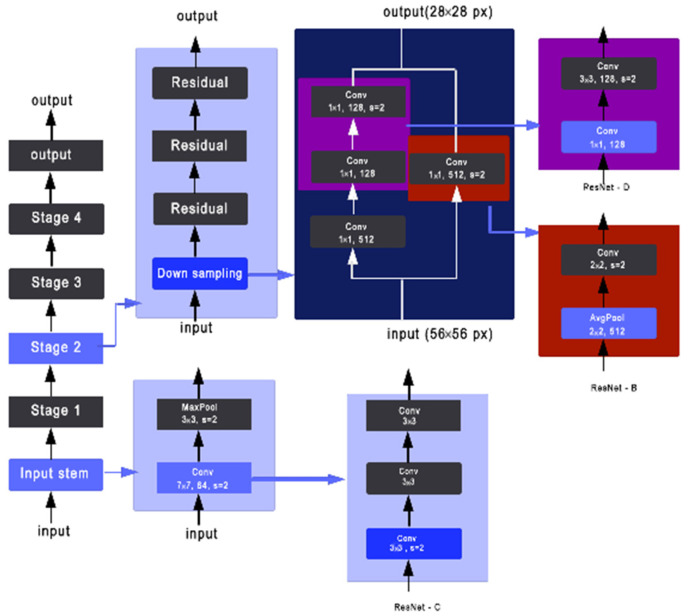
Key aspects of the xResNet50 architecture.

Tong He [27] introduced XResNet with three different methods to improve the three separate convolutional steps that are presented in the ResNet structure. As shown in Figure 3 and Figure 4, ResNet-B moves the stride (2,2) to the second convolution and keeps a stride of 1 for the first layer [27]. ResNet-C removes the 7 × 7 convolution and replaces it with three consecutive 3 × 3 convolutions. Finally, ResNet-D replaces the 1 × 1 convolution of stride (2,2) with a (2,2) average pooling layer of stride (2,2) followed by a 1 × 1 convolution layer [28].

**Figure 5:**
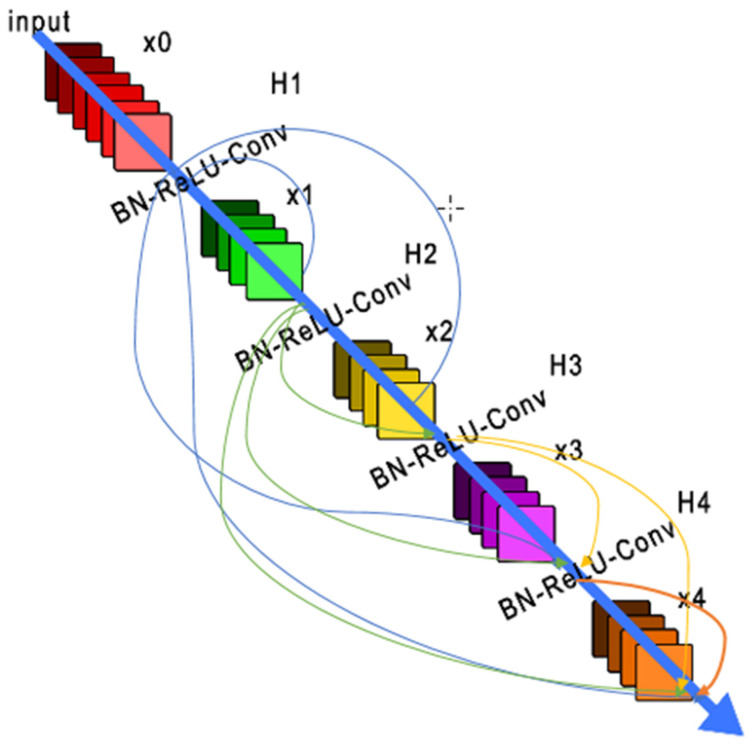
A 5-layer dense block with a growth rate of k = 4. Each layer takes all preceding feature-maps as input.

The designer presented a dense convolutional network (DenseNet), an architecture that distills the complexity of other CNN learners into a simple connectivity pattern, so that with this system, the maximum information will flow between layers in the network and all layers will be directly connected with each other. To preserve the feed-forward nature, each layer obtains additional inputs from all preceding layers and passes them on to all subsequent layers. Figure 5 shows this layout schematically. In contrast to ResNets, we never combine features through summation before they are passed into a layer; instead, we combine features by concatenating them. Hence, the layer has inputs consisting of the feature maps of all the preceding convolutional blocks. Its own feature maps are passed on to all subsequent layers. This introduces connections in an L-layer network instead of just an L-layer network, as in traditional architectures. Because of its dense connectivity pattern, we refer to our approach as “Dense Convolutional Network” (DenseNet) [28].

The AlexNet has eight layers with learnable parameters. The model consists of five layers with a combination of max pooling followed by three fully connected layers, and they use Relu activation in each of these layers except the output layer [9]. 

Ref. [9] found out that using the relu as an activation function accelerated the speed of the training process by almost six times. They also used the dropout layers to prevent their model from overfitting. Furthermore, the model is trained on the ImageNet dataset. The ImageNet dataset has almost 14 million images across a thousand classes.

**Figure 6:**
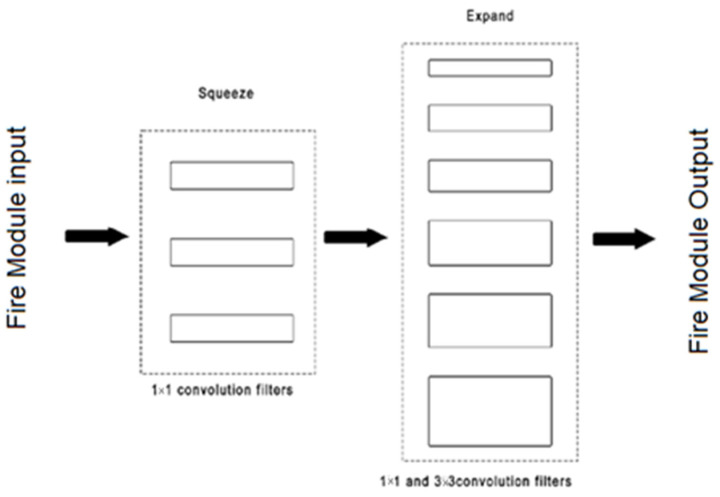
Micro-architectural view: convolution filters organization in the fire modules [30].

The SqueezeNet [29] is a smaller CNN architecture that uses fewer parameters while maintaining competitive accuracy. Several strategies are employed on the CNN basis to design the SqueezeNet: (1) replace 3 × 3 filters with 1 × 1 filters, (2) decrease the number of input channels to 3 × 3 filters, and (3) downsample late in the network so that the convolution layers have large activation maps. The SqueezeNet is comprised mainly of Fire modules that are squeeze convolution layers with only 1 × 1 filters. These layers are then fed into an expand layer, which has a mix of 1 × 1 and 3 × 3 convolution filters, as shown in Figure 6. 

**Figure 7:**
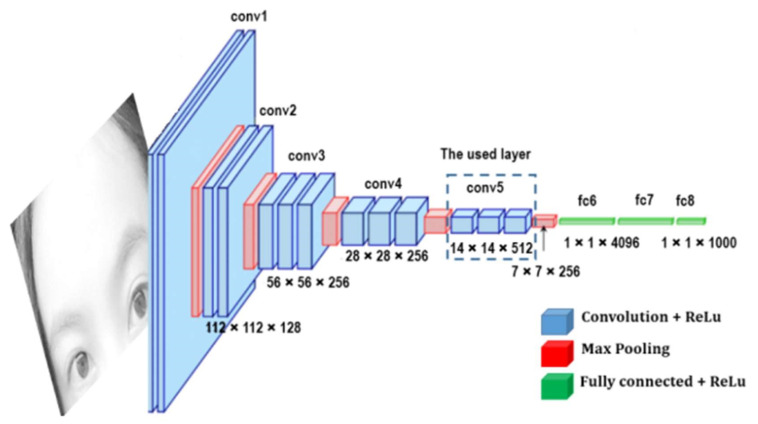
Architecture of VGG16 [17].

VGG16 is a convolution neural network (CNN) architecture that was used to win the ILSVR (ImageNet) competition in 2014. It is regarded as one of the best vision model architectures available to date. Its structure is shown in Figure 7. The most unique thing about VGG16 is that instead of having a large number of hyper-parameters, they focused on having convolution layers of a 3 × 3 filter with a stride 1 and always used the same padding and maxpool layer of a 2 × 2 filter with a stride 2. It follows this arrangement of convolution and max pool layers consistently throughout the whole architecture. In the end, it has two FC (fully connected layers), followed by a softmax for output. The 16 in VGG16 refers to it 16 layers that have weights. This network is pretty large, and it has about 138 million (approximately) parameters [29]. 

**Figure 8:**
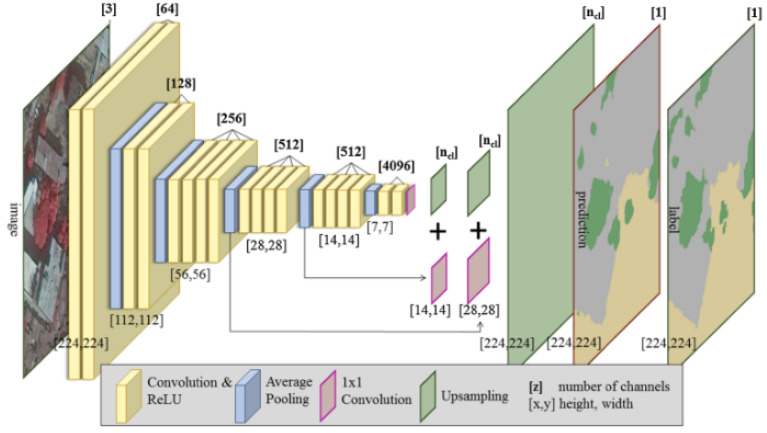
Structure of VGG19 [16].

The architecture of the FCN-VGG19, shown in Figure 8, is adapted from [29], which learns to combine high-level information with fine-level information using skips from the third and fourth pooling layers. The hidden layers are equipped with rectified linear units (ReLUs), and the number of channels for the convolutional layers increases with the depth of the network. During training, the input image is a fixed size of 224 × 224 pixels, while the receptive fields for all filters are 3 × 3 pixels throughout the whole network. This configuration allows the FCN (fully convolutional networks) to learn approximately 140 million parameters. Prediction is performed using upsampling layers with four channels for all classes [ncl] in the reference data. Upsampling layers are fused with 1 × 1 convolutions of the third and fourth pooling layers with the same channel dimension [x,y,ncl]. The final upsampling layer predicts fine details using fused information from the last convolutional layer, with the third and fourth pooling layers being upsampled at stride 8.

Table 1 shows a summary of the described CNN learning methods.

The last parameter that should be considered in the learning models is time complexity. The quantity of fundamental operations, such as multiplications and summations, that an algorithm performs determines its time complexity. Typically, the time complexity is stated as a function of the size n of the input. The size of the input, or n, which indicates how many items are taken into account for the input, must first be known in order to determine the time complexity. How many operations are made in relation to the amount of the input is the second parameter that affects time complexity (number of epochs). so the linear time complexity is O(2 × n) = O(n).

The following table compares the structure and different working conditions of each of the learning systems considered in this study.

## 3. Dataset

Traffic signs seen from a vehicle present themselves in various conditions that make it difficult to recognize them, such as different distances (low image resolution), lighting, vandalism, or even some obstacles, such as leaves on trees, which can compromise visibility. With this in mind, it is necessary to use a database with multiple images that can cover these different conditions during training [31].

The database used is called “The German Traffic Sign Recognition Benchmark”, or GTSRB, and was used for neural network competitions in 2011. It has 43 different classes of signs found in Germany and about 50,000 images in total. Due to its large number of images, it is possible to achieve very good results, but it also has some defects that will be discussed later [32].

The images for the model were loaded and prepared with fastai tools so that each class was divided between images and a label, which, in this case, is the name of the folder where the images of each class are located. Folders have been renamed from the original basis so that the results can be presented more clearly. In addition, the images were randomly divided into training images and validation images at a ratio of 4 training images to each validation image, resulting in approximately 40 thousand for training and 10 thousand for validation, and, finally, resized for training [31].

## 4. Models and Methods

**Figure 9:**
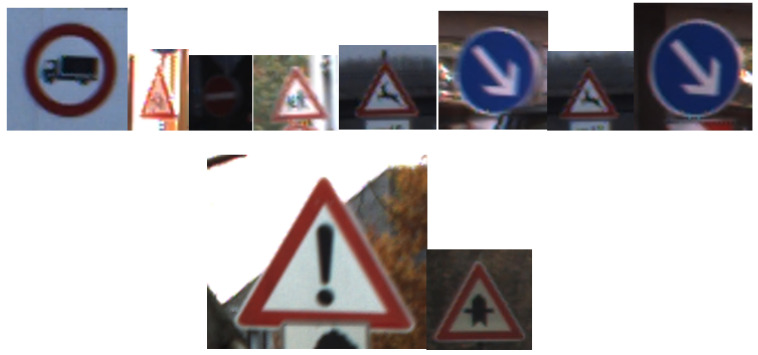
Some of the images from the dataset.

The recognition model was trained several times by different types of CNN-learners, observing the accuracy at the end of the training, the amount of processing used, and the time to perform the training. Some examples of the employed dataset are shown in Figure 9. The learner models used in this article and whose results will be reviewed and compared, are ResNet 18, 34, and 50; DenseNet 121, 169, and 201; Vgg 16_bn and Vgg19_bn; SqueezeNet1_0 and SqueezeNet1_1; and AlexNet and XresNet50.

**Figure 10:**
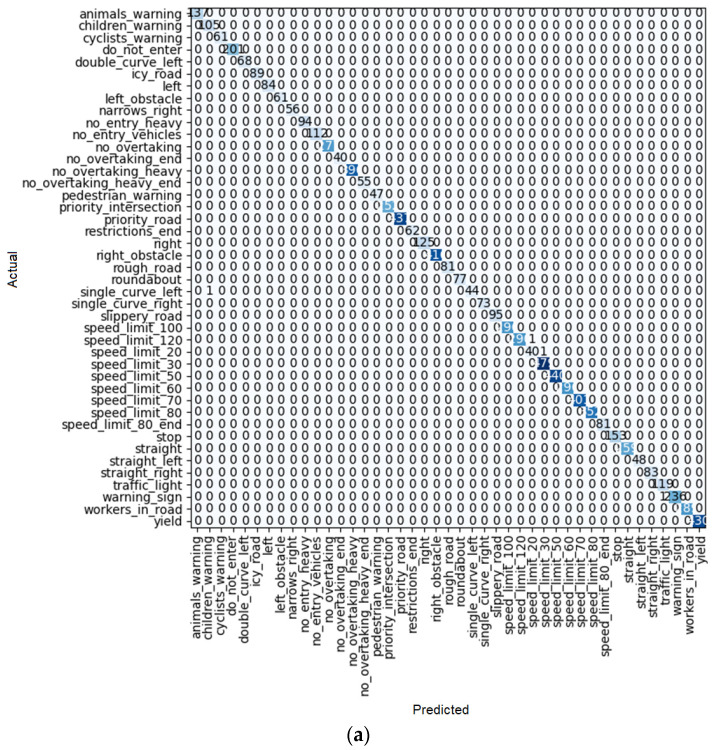

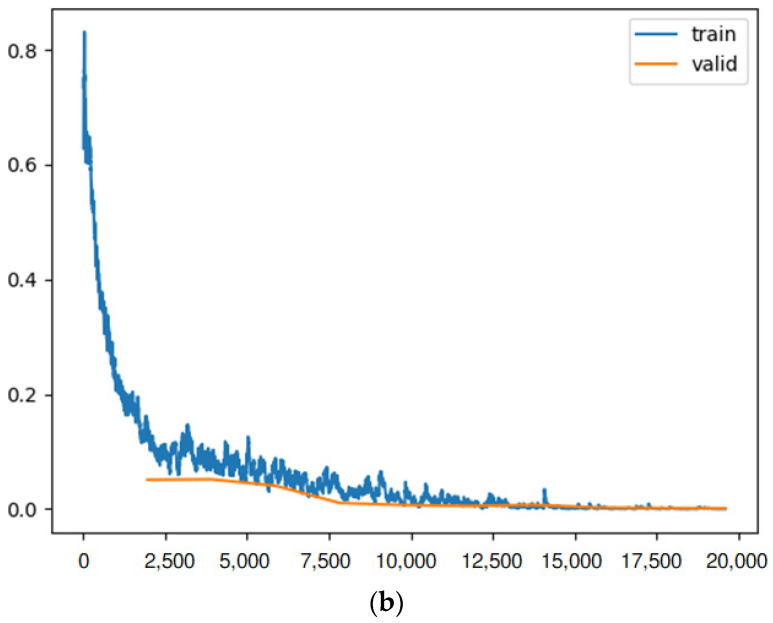
(**a**) Confusion matrix and (**b**) loss rate during training with ResNet18.

To find the most efficient way to train the model, some elements were changed: the size of each training batch, the number of training iterations (fine tuning), and the number of layers (for example, for the ResNet model), so that the loss rate (similar to Figure 10b) for training and validation at the end of training is as close as possible, indicating training with little overfitting or underfitting. In addition, the outcome of each learner system is displayed in a confusion matrix similar to Figure 10a. For each method of learning, there is a table that shows the result of train loss, valid loss, accuracy, error rate, and the desired time of training for each epoch.

The results can vary due to some other factors, the main ones being the random initialization of the values and the scrambling of the training data implemented by the fastai algorithm. However, it was possible to notice some patterns in the different trainings performed, where changing values considerably improved the result to the point of stagnation.

## 5. Result

The batch size, number of iterations, training model, and number of layers were changed while training the recognition model multiple times. Accuracy was measured at the end of each training cycle, along with the processing power and training time required. Large batches were shown to take less time, use more computer memory, but lose a significant amount of accuracy after training.

The best outcome was 16, whereas the largest batch value tested was 128, where lower values did not indicate greater accuracy, and longer training sessions did not result in any appreciable gains.

### 5.1. ResNet

#### 5.1.1. ResNet 18

The first note that should be considered is that in the number of iterations, up to four iterations, the training loss rate decreases considerably; from 4 to 5, it presents a considerable increase, but again after iteration 5, it decreases again. 

Table 2 and Figure 9 shown the results of the ResNet 18 CNN learner.

As shown in Figure 10a, after 10 iterations, only one of the ResNet 18 predictions had an error, and it was the single-curve-left sign predicted as the children’s warning sign. For the others, all the predictions were correct.

#### 5.1.2. ResNet 34

**Figure 11:**
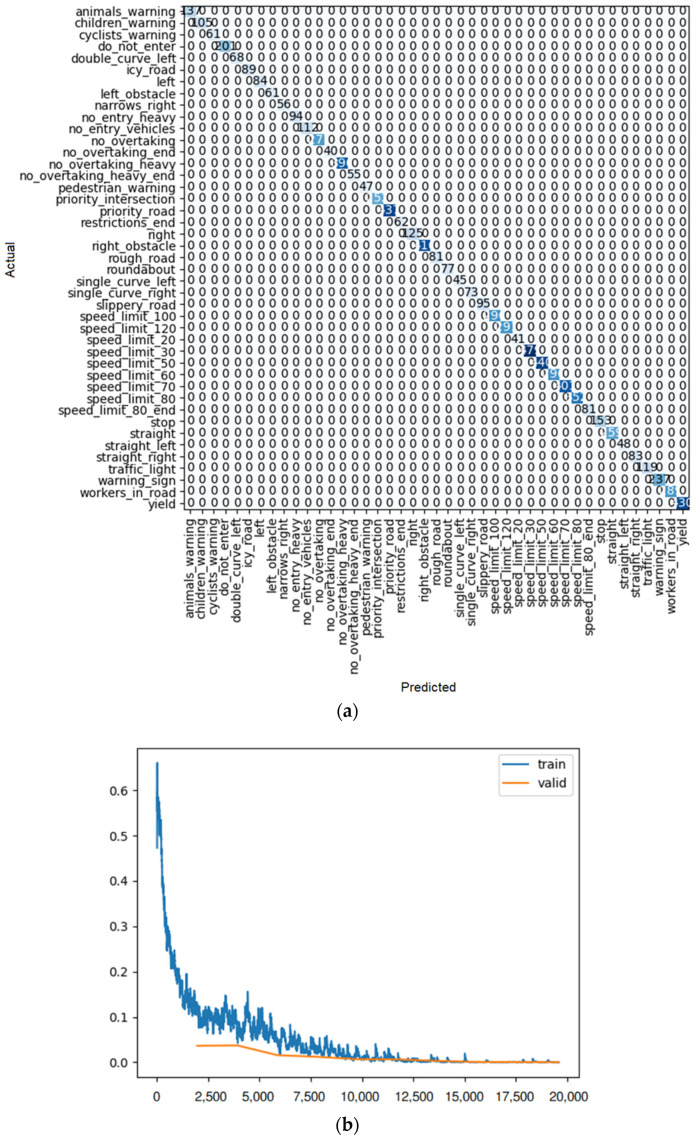
(**a**) Confusion matrix and (**b**) loss rate during training with ResNet 34.

The number of iterations in ResNet 34 is similar to ResNet 18, which has 10 iterations. Used in fine tuning, it was observed that up to 3 iterations the training loss rate decreased considerably; from 3 to 4, it presented an increase, but again after iteration 4, it totally decreased again. So as a very simple result, it can be concluded that, in this case compared with ResNet 18, the error tends to zero faster than in the other case. The results are shown in Table 3 and Figure 11.

#### 5.1.3. ResNet 50

**Figure 12:**
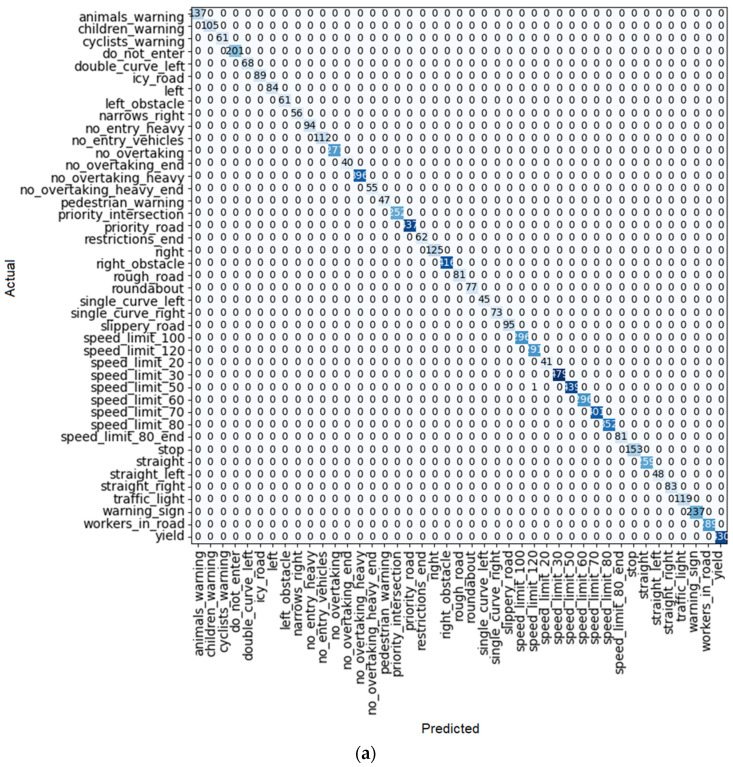

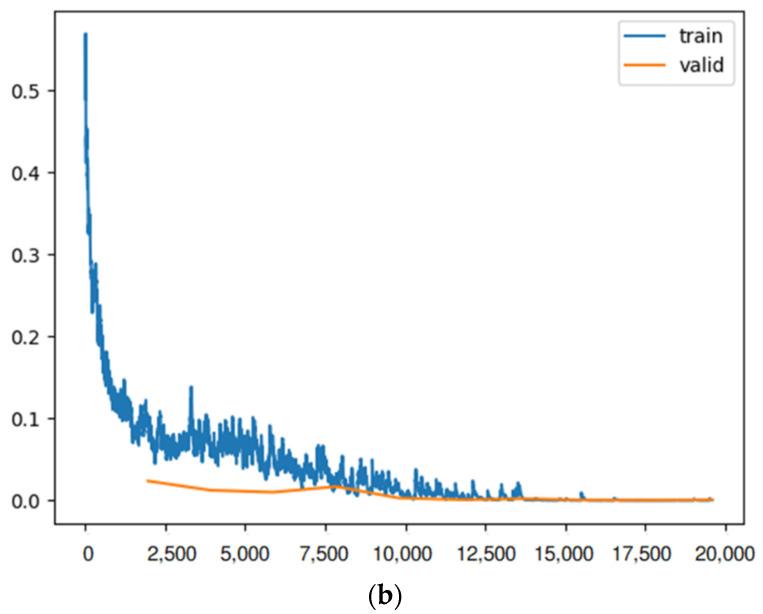
(**a**) Confusion matrix and (**b**) loss rate during training with ResNet 50.

Among the ResNet learning models, ResNet 50 has the best performance because its train loss trend is completely downward. The results are shown in Table 4. The only place where there is some improvement in this process is in the last iteration, which has increased somewhat. Moreover, as shown in Figure 12a, only one error occurred in the detection of traffic signs.

### 5.2. Squeezenet

**Figure 13:**
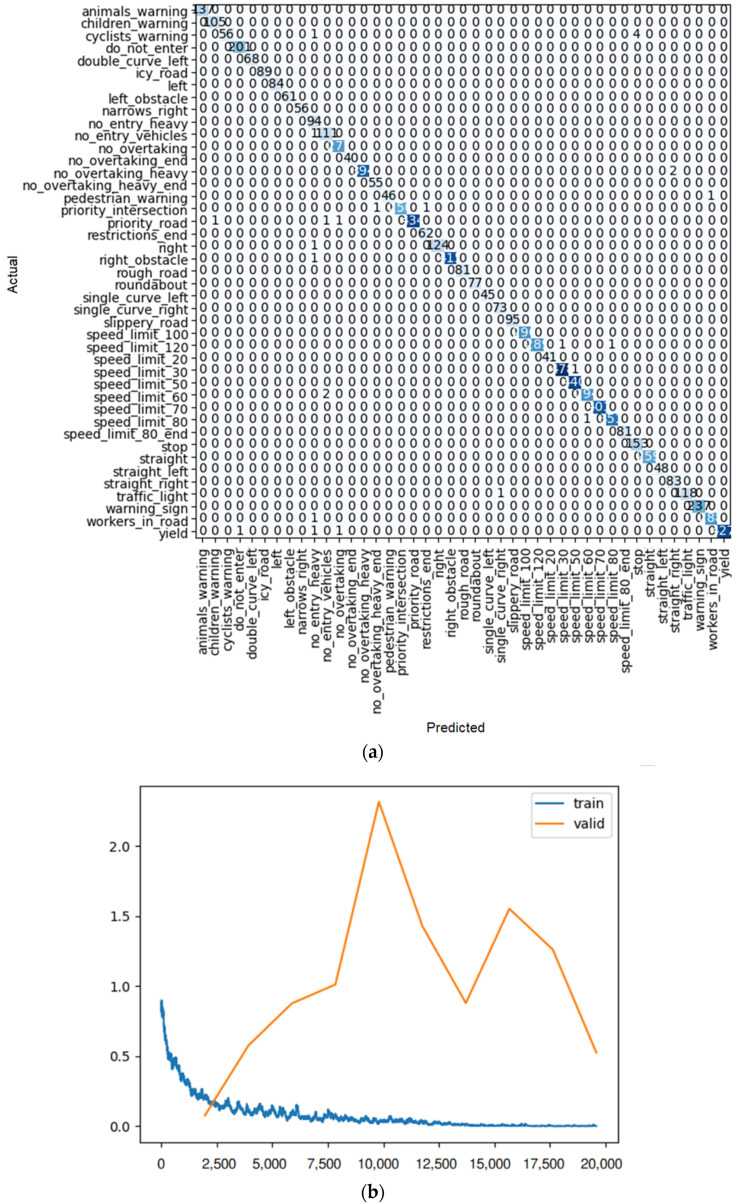
(**a**) Confusion matrix and (**b**) loss rate during training with SqueezeNet1_0.

**Figure 14:**
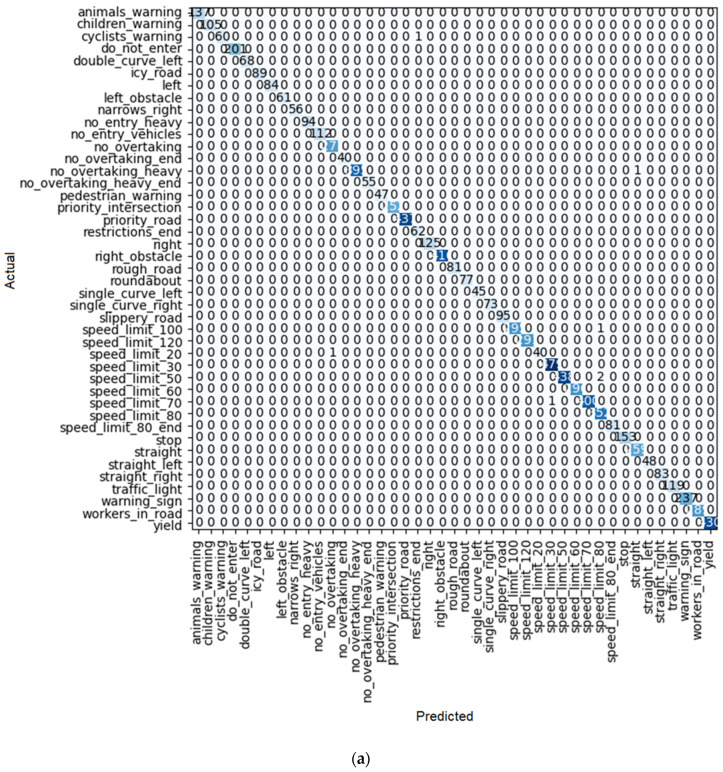

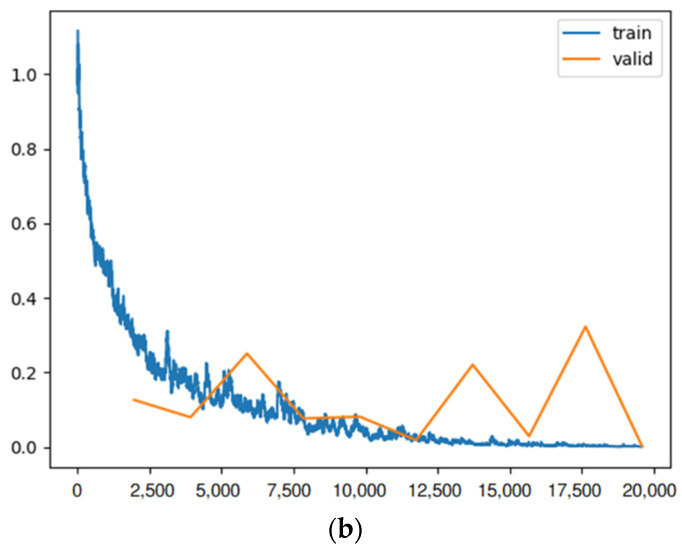
(**a**) Confusion matrix and (**b**) loss rate during training with SqueezeNet1_1.

About the train loss of both SqueezeNet 1_0 and SqueezeNet 1_1, both of them have a downward trend, just as the accuracy has an upward trend. However, the problem starts when we examine the valid loss and error rate. As shown in Table 5 and Table 6, as well as Figure 13b and Figure 14b, a lot of fluctuation can be seen in these two parameters. As a result, these two cases are not suitable for this purpose because they had weak validation results. The proof of this claim can be seen in Figure 13a and Figure 14b. This is because the system errors in detecting traffic signs are very high.

### 5.3. VGG

One of the best methods studied so far is VGG. This issue can be seen further.

#### 5.3.1. VGG16_bn

**Figure 15:**
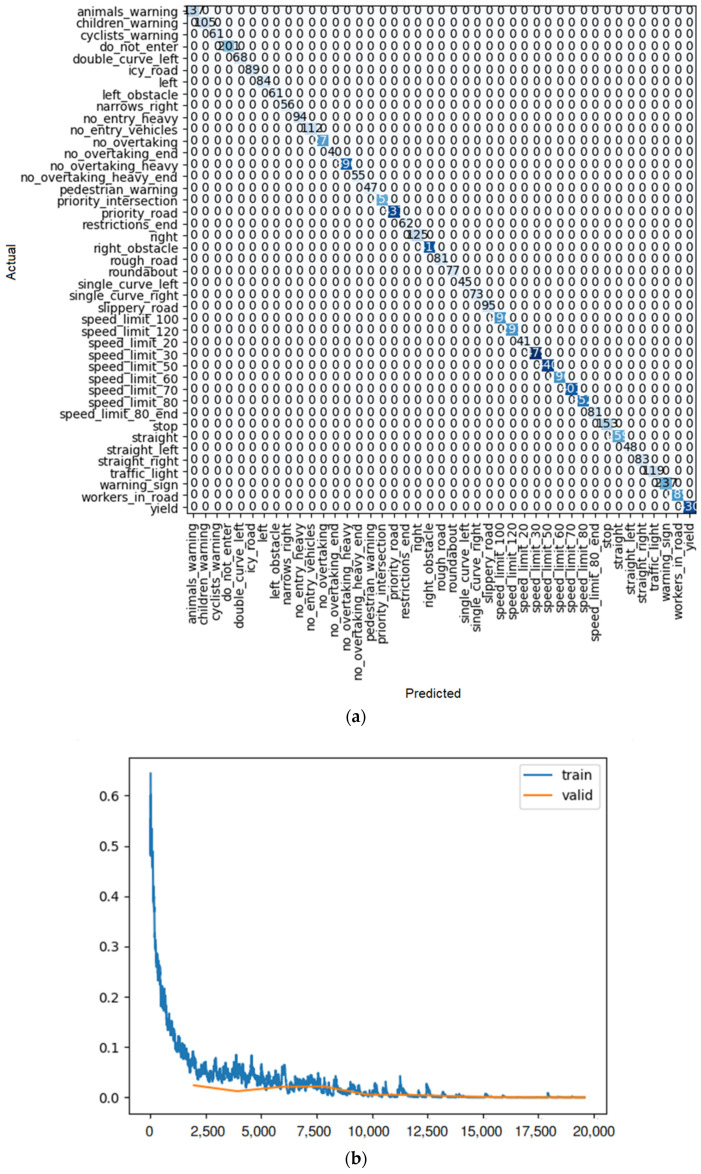
(**a**) Confusion matrix and (**b**) loss rate during training with VGG16_bn.

In the case of the VGG16_bn method, as shown in Table 7 and Figure 15, the train loss trend is completely downward, and the accuracy of the system reaches 100% in the last epoch. On the other hand, valid loss reached a very low number of 0.000127, and the error rate completely reached zero. As a result, this is one of the methods that has the ability to be introduced as the best method at the end.

#### 5.3.2. VGG19_bn

**Figure 16:**
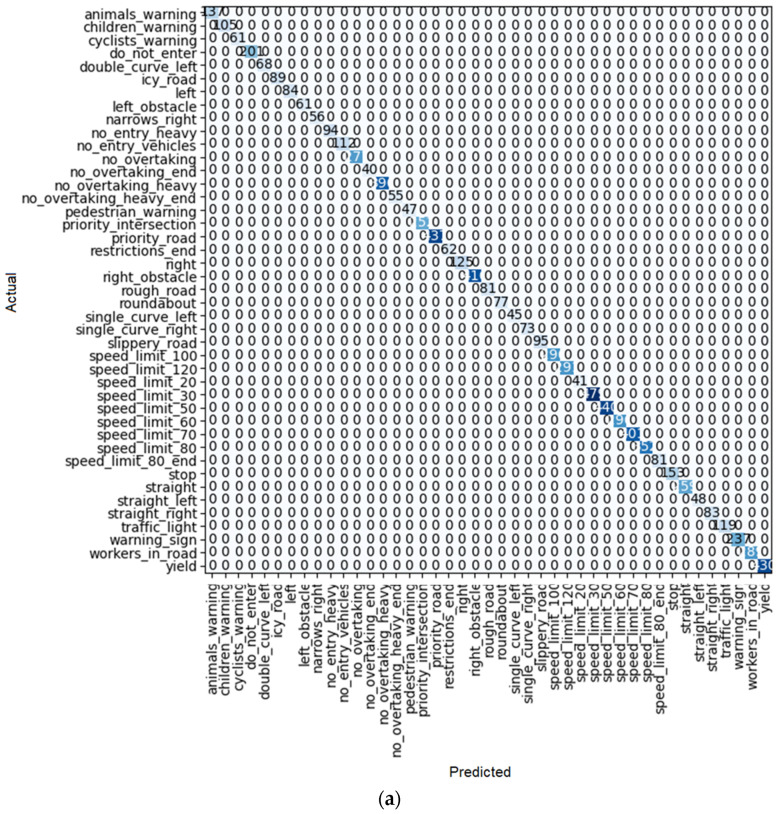

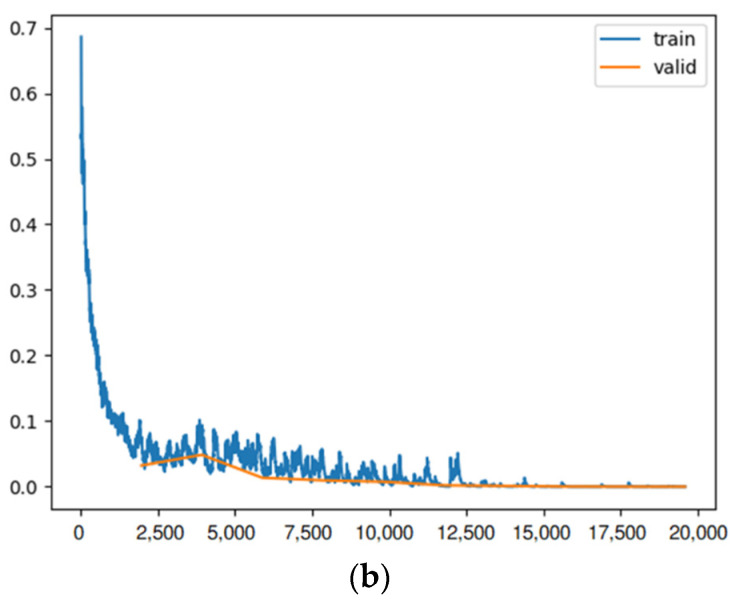
(**a**) Confusion matrix and (**b**) loss rate during training with VGG19_bn.

Another method from the VGG group that was investigated is VGG19_bn. As shown in Table 8, the train loss in this method was 0.00004 in the last epoch, its accuracy reached 100 percent in the eighth epoch, and the error rate reached 0 in this epoch. The only negative point about this method compared to the previous one is that the time required for each epoch is a bit longer. Other data is shown in Figure 16.

### 5.4. XresNet 50

**Figure 17:**
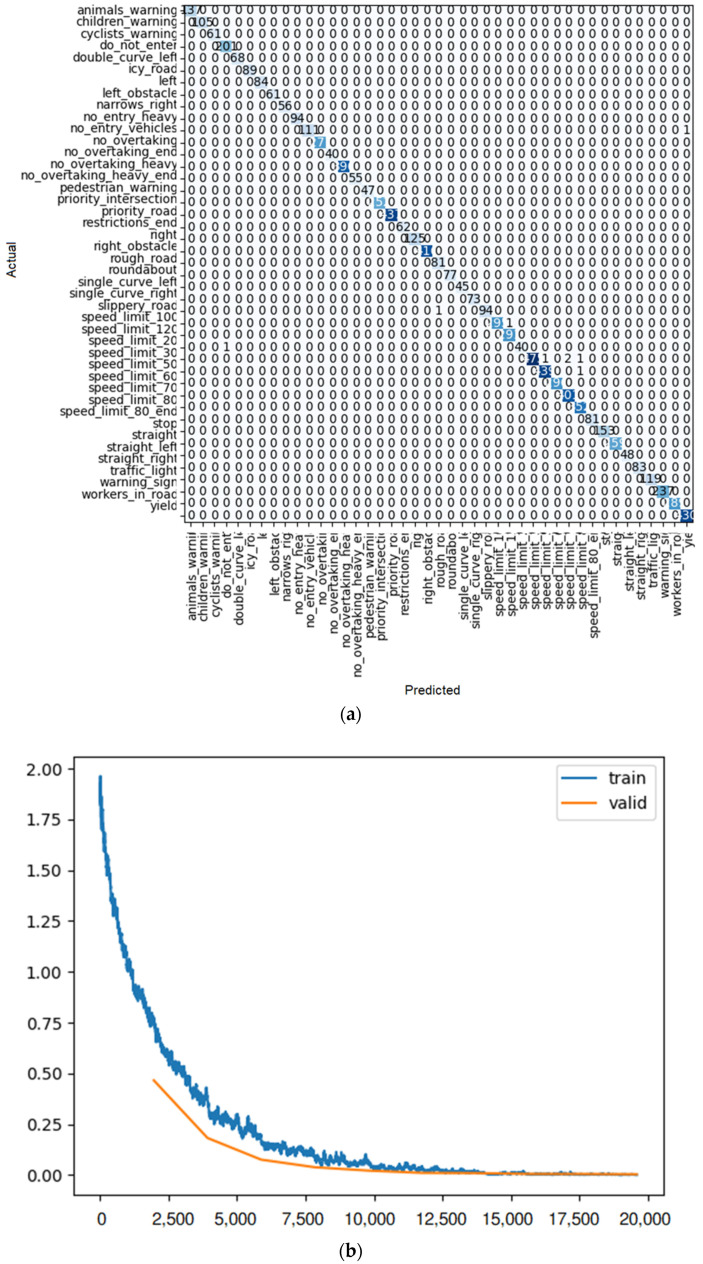
(**a**) Confusion matrix and (**b**) loss rate during training with XResNet5.

Another method that has been investigated is XresNet 50. This method is one of the most widely used for image classification. As shown in Table 9, in this method, the valid loss has reached 0.000869 in the last epoch. Moreover, by considering Figure 17a, a large error can be seen in the detection of traffic signs. Another important parameter, which is very important, is the accuracy, which reached 99.8852% in this method. As a result, if we compare with the previous methods, we realize that this model cannot be among the candidates for the best investigated methods.

### 5.5. Densenet

**Figure 18:**
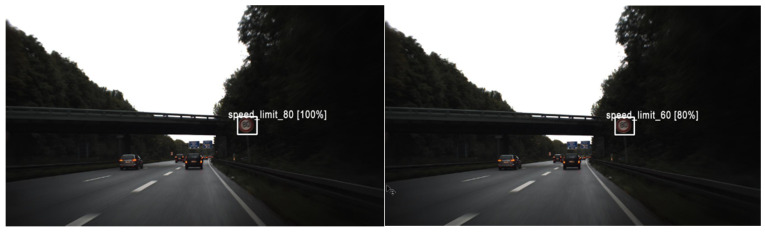
Comparing the performance of one of the wrong diagnoses by model DenseNet 169 and DenseNet 201, which was correctly diagnosed by model DenseNet 121.

**Figure 19:**
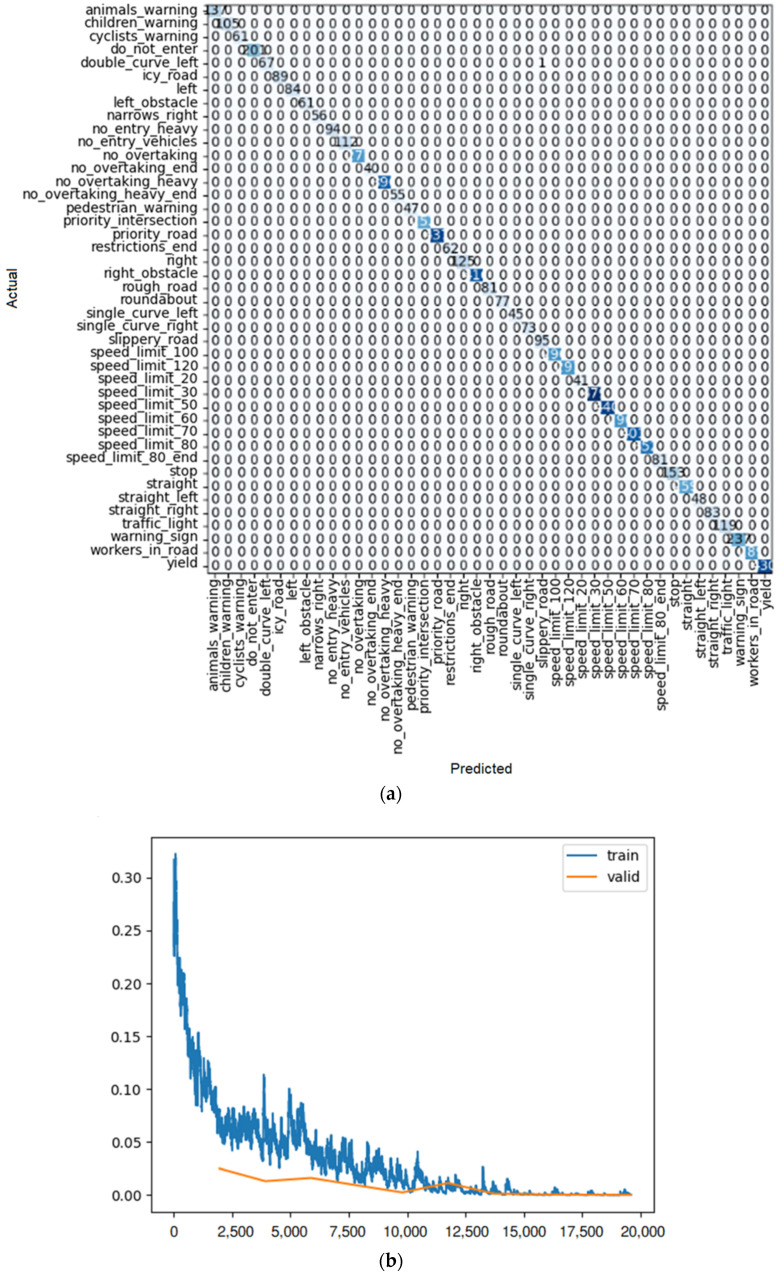
(**a**) Confusion matrix and (**b**) loss rate during training with DenseNet 121.

**Figure 20:**
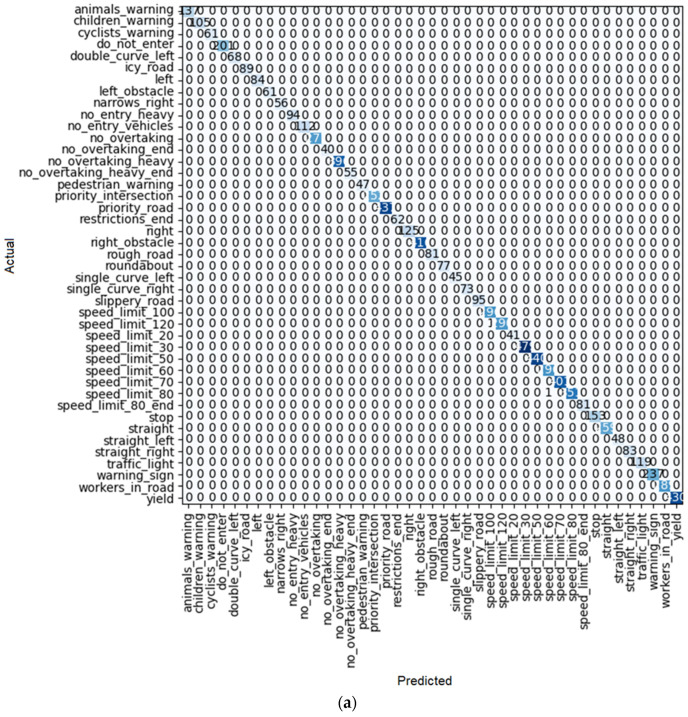

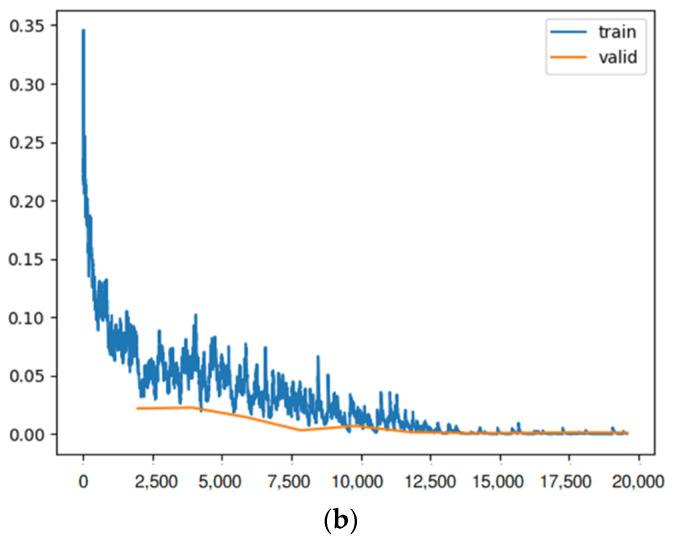
(**a**) Confusion matrix and (**b**) loss rate during training with DenseNet 169.

**Figure 21:**
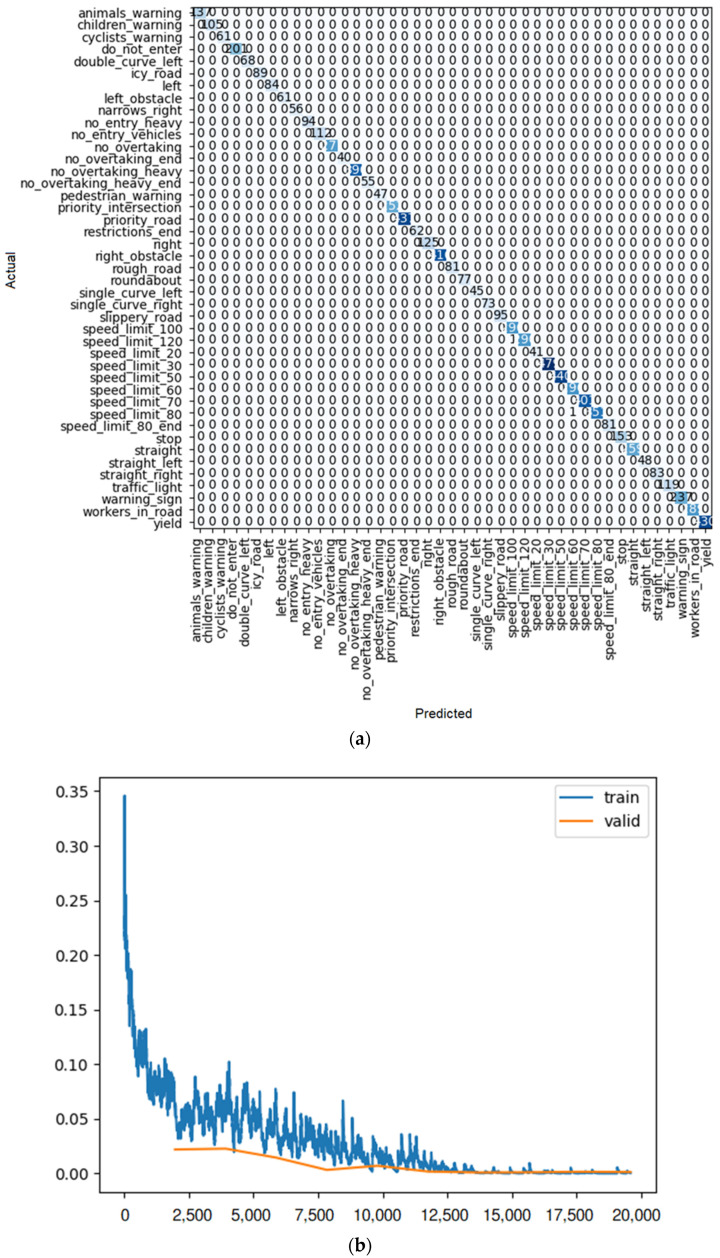
(**a**) Confusion matrix and (**b**) loss rate during training with DenseNet 201.

By looking at the results of the DenseNet group, it can be seen that this group is also one of the best for detecting traffic signs. As seen in Table 10, Table 11 and Table 12, train loss experiences a downward trend with low volatility in these models. Moreover, the error rate in DenseNet 121, 169, and 201 reaches 0.000128, 0.000255, and 0.000510, respectively. However, in the case of DenseNet 121, there is no error in detection. For example, one of these errors is shown in Figure 18. As shown in this figure, there is a traffic sign with a speed limit of 80 km/h, which is correctly recognized by model DenseNet 121, but by models DenseNet 169 and DenseNet 201, it is wrongly recognized as a speed limit of 60 km/h. The superiority of DenseNet 121 over DenseNet 169 and 201 is clearly evident in the parameters of the table. In DenseNet 169 and 201, an error in detecting traffic signs is also observed, as shown in Figure 19a, Figure 20a, and Figure 21a. 

### 5.6. AlexNet

**Figure 22:**
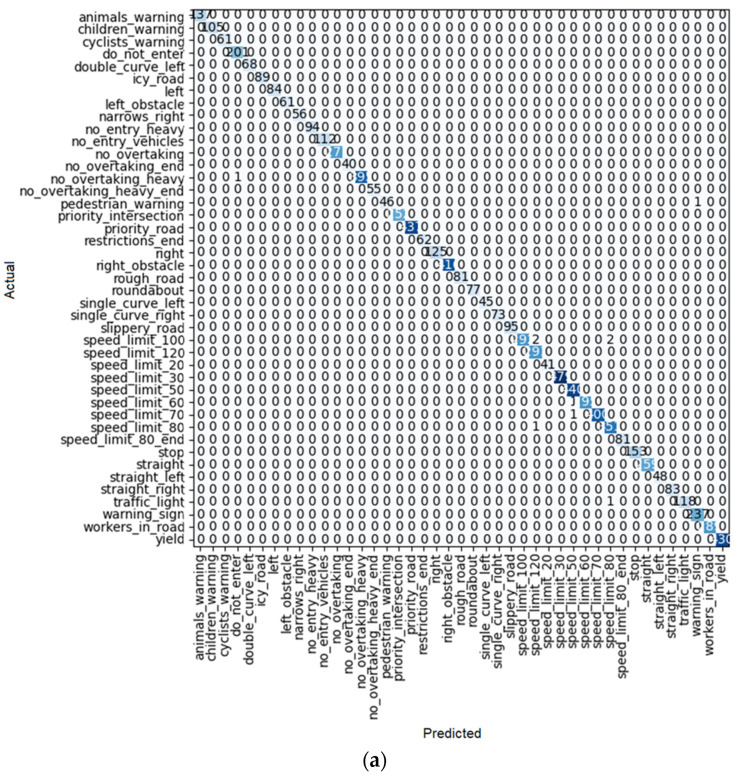

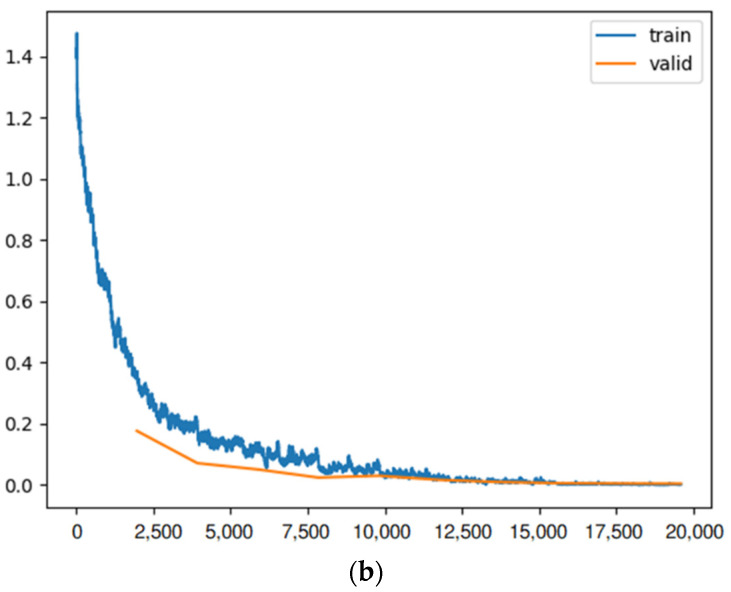
(**a**) Confusion matrix and (**b**) loss rate during training with AlexNet.

The last method that is examined in this article is AlexNet. A very positive point that can be seen in Table 13 of this method is the time required for each epoch. This time is only 12 min on average. On the other hand, if we have a tradeoff between the time required for each epoch, train loss, and accuracy, we realize that this system can be introduced as one of the best methods. The results are shown in Figure 22.

On the other hand, all the parameters of this system follow a completely uniform trend without fluctuations during different epochs, which is another reason for the goodness of this method.

## 6. Comparison

As shown in the above figures and tables, models SqueezeNet1_0 and SqueezeNet1_1 could not be used for this project because they did not learn correctly, and therefore they are not used in comparison. For the other methods, we use the last epoch of each learning model to compare them with each other.

As shown in the above table, and by considering that each iteration is equivalent to 1960 batches for training and 491 for validation, if maximum accuracy and minimum system error are important, the best results can be VGG16_bn and VGG19_bn. However, for these two models, the time required is the longest compared to other systems. But if less training time is desired with acceptable accuracy and error, AlexNet is the best option. As shown in Table 14, after ten epochs, the accuracy of the system is 0.998725 and the error rate is just 0.001275, very close to zero. Moreover, with these parameters, the time needed for the training for the last epoch is just about 13 min.

## 7. Conclusions 

As a result, this article demonstrates that in the same situation, with the same data set structure and processor (Intel(R) Core(TM) i5-4300M CPU @ 2.60 GHz, 2.59 GHz), we can compare the mentioned models and find the best training model for a traffic sign detection system.

So, as shown in Table 14, the best system with acceptable error and accuracy and the shortest time required for training is the VGG19_bn model.

Two very important limitations that can be seen in the results are: (1) the speed of CNN learning systems and (2) the problem of classification with different positions. As for the second problem, when there is some tilt or rotation in the images, CNNs usually have trouble classifying them.

After further study and after finding the best method, in future studies this method can be examined online and on video in real time, exactly what is needed in driverless cars.

Each classifier relies on its own properties, which are determined by the specific classifier. The optimal parameter values depend on the scenario in which the classifier is to be applied. This study showed that it is not always possible to set the parameters accurately when applying classifiers to different data sets. If we study the future, firstly, we will work with different learning models similar XceptionV2, MobileNetV2, also we will likely do network searches to find different parameter values and then choose a parameter that is the best match. This maximizes accuracy because it is not guaranteed to find the absolute optimal value for a given classifier in a given data set, but it constitutes a good approximation.

## Figures and Tables

**Table 1:** Comparison of structure and input in different CNN learning models.

Model	Size	Top-1/Top-5 Error	# Layer	Model Description
AlexNet	227 × 227	41.00/18.00	8	5 conv + fc layers
VGG 16	224 × 224	28.07/9.00	16	13 Conv + 3 fc layers
VGG 19	224 × 224	27.30/9.33	19	16 Conv +3fc layers
ResNet 50	224 × 224	22.85/6.71	50	49 conv + 1 fc layers
ResNet 18	224 × 224	20.47/5.25	18	17 conv + 1 fc layers
ResNet 32	224 × 224	21.53/5.60	34	33 conv + 1 fc layers
XResNet 50 (C)	224 × 224	-	50	1 conv 3 × 3, s = 2 +2 conv 3 × 3 +1 MaxPool 3 × 3, s = 2
DenseNet 121	224 × 224	25.35/7.83	125	1 7 × 7 conv + 58 3 × 3 conv + 61 1 × 1 conv + 4 AvgPool 1 fc Layers
DenseNet 169	224 × 224	24.00/7.00	169	7 × 7 stride 2 Conv Layer followed by a 3 × 3 stride-2 MaxPooling layer
DenseNet 201	224 × 224	22.8/6.43	201	7 × 7 stride 2 Conv Layer followed by a 3 × 3 stride-2 MaxPooling layer
SqueezeNet 1-0	227 × 227	41.90/19.58	18	1 Conv layer + 8 fire modules + 10 conv layer
SqueezeNet 1-1	227 × 227	41.81/19.38	18	1 Conv layer + 8 fire modules + 10 conv layer

**Table 2:** Results of the ResNet 18.

Epoch	Train Loss	Valid Loss	Accuracy	Error Rate	Time
0	0.858337	0.478402	0.847979	0.152021	42:37
0	0.142488	0.051000	0.982910	0.017090	46:55
1	0.089376	0.051660	0.985461	0.014539	51:24
2	0.072999	0.040365	0.989542	0.010458	51:32
3	0.034141	0.010321	0.996684	0.003316	52:10
4	0.010182	0.006904	0.997832	0.002168	52:41
5	0.015726	0.005160	0.998597	0.001403	51:56
6	0.008586	0.007243	0.997959	0.002041	51:01
7	0.000352	0.002578	0.998980	0.001020	51:11
8	0.000050	0.001568	0.999362	0.000638	51:17
9	0.000053	0.001247	0.999490	0.000510	52:05

**Table 3:** Results of the ResNet 34.

Epoch	Train Loss	Valid Loss	Accuracy	Error Rate	Time
0	0.656895	0.345476	0.890065	0.109935	1:09:47
0	0.118535	0.036422	0.989415	0.010585	1:40:23
1	0.052004	0.037212	0.990817	0.009183	1:38:41
2	0.045372	0.015638	0.995919	0.004081	1:34:24
3	0.024831	0.012060	0.997322	0.002678	1:32:30
4	0.025713	0.006691	0.998215	0.001785	1:34:21
5	0.018851	0.006416	0.998342	0.001658	1:35:12
6	0.004856	0.002991	0.999107	0.000893	1:32:53
7	0.001296	0.000732	0.999745	0.000255	1:30:48
8	0.001313	0.000481	0.999617	0.000383	1:34:05
9	0.000018	0.000238	1.000000	0.000000	1:35:50

**Table 4:** Results of the ResNet 50.

Epoch	Train Loss	Valid Loss	Accuracy	Error Rate	Time
0	0.494744	0.228529	0.926668	0.073332	2:04:54
0	0.093774	0.023691	0.992858	0.007142	2:43:09
1	0.055501	0.012457	0.995791	0.004209	2:40:10
2	0.053681	0.010073	0.996939	0.003061	2:38:37
3	0.018222	0.016950	0.993878	0.006122	2:38:05
4	0.017393	0.003013	0.999107	0.000893	2:35:11
5	0.006129	0.000881	0.999745	0.000255	2:28:34
6	0.001446	0.002066	0.999490	0.000510	2:32:31
7	0.000552	0.000578	0.999745	0.000255	3:00:53
8	0.000033	0.000318	0.999872	0.000128	3:02:22
9	0.000536	0.000964	0.999745	0.000255	3:18:05

**Table 5:** Results of the SqueezeNet 1-0.

Epoch	Train Loss	Valid Loss	Accuracy	Error Rate	Time
0	0.914806	0.471145	0.841602	0.158398	16:19
0	0.185827	0.066723	0.980232	0.019768	30:13
1	0.114141	0.135726	0.985334	0.014666	30:24
2	0.081658	0.377006	0.980870	0.019130	30:22
3	0.039321	0.065290	0.992220	0.007780	29:52
4	0.043154	0.705668	0.994006	0.005994	29:54
5	0.013735	0.314177	0.993496	0.006504	30:21
6	0.008455	0.249811	0.996301	0.003699	27:08
7	0.011575	0.512464	0.997322	0.002678	26:54
8	0.002557	0.397922	0.997704	0.002296	27:04
9	0.002186	0.592725	0.996557	0.003443	26:49

**Table 6:** Results of the SqueezeNet 1-1.

Epoch	Train Loss	Valid Loss	Accuracy	Error Rate	Time
0	1.092300	0.615839	0.799643	0.200357	08:38
0	0.294047	0.126106	0.961867	0.038133	17:22
1	0.157563	0.079483	0.972325	0.027675	16:59
2	0.112692	0.250555	0.974238	0.025762	17:01
3	0.085473	0.076384	0.987757	0.012243	17:31
4	0.053619	0.081055	0.992986	0.007014	16:57
5	0.027114	0.018042	0.996429	0.003571	16:51
6	0.010796	0.220587	0.996684	0.003316	16:51
7	0.010787	0.028796	0.998852	0.001148	17:09
8	0.003092	0.322757	0.996939	0.003061	17:06
9	0.000727	0.002551	0.999107	0.000893	16:32

**Table 7:** Results of the SqueezeNet1-1.

Epoch	Train Loss	Valid Loss	Accuracy	Error Rate	Time
0	0.624660	0.282934	0.907537	0.092463	3:05:02
0	0.074642	0.023860	0.992475	0.007525	4:11:43
1	0.054696	0.015138	0.996301	0.003699	3:57:00
2	0.022167	0.020754	0.995791	0.004209	3:49:24
3	0.021000	0.021600	0.996046	0.003954	4:46:25
4	0.002759	0.005120	0.999107	0.000893	4:21:58
5	0.004077	0.004919	0.998725	0.001275	4:12:50
6	0.000342	0.002219	0.999235	0.000765	4:17:23
7	0.000396	0.000540	0.999872	0.999128	4:15:03
8	0.000033	0.000209	0.999872	0.000128	4:14:16
9	0.000009	0.000127	1.000000	0.000000	4:36:52

**Table 8:** Results of the VGG19_bn.

Epoch	Train Loss	Valid Loss	Accuracy	Error Rate	Time
0	0.741779	0.309815	0.901288	0.098712	5:19:59
0	0.074820	0.032293	0.991328	0.008672	6:03:03
1	0.076397	0.048556	0.989160	0.010840	5:50:49
2	0.024736	0.013608	0.997067	0.002933	6:41:47
3	0.051280	0.009662	0.997194	0.002806	6:51:02
4	0.006818	0.007519	0.998342	0.001658	5:49:44
5	0.000993	0.002135	0.999490	0.000510	5:40:14
6	0.000986	0.001120	0.999872	0.000128	5:45:10
7	0.002016	0.000211	0.999872	0.000128	5:28:46
8	0.000020	0.000031	1.000000	0.000000	5:29:33
9	0.000004	0.000002	1.000000	0.000000	5:25:54

**Table 9:** Results of the XResNet50.

Epoch	Train Loss	Valid Loss	Accuracy	Error Rate	Time
0	0.011813	1.375518	0.603750	0.396250	2:05:28
0	0.738159	0.465996	0.866854	0.133146	2:33:33
1	0.372449	0.182658	0.945543	0.054457	2:29:01
2	0.164344	0.076088	0.975258	0.024742	2:32:48
3	0.087024	0.038646	0.986354	0.013646	2:31:17
4	0.065194	0.022141	0.993113	0.006887	2:33:16
5	0.043767	0.010952	0.996812	0.003188	2:40:05
6	0.015639	0.009900	0.997832	0.002168	2:34:36
7	0.004813	0.005847	0.998342	0.001275	2:37:55
8	0.001854	0.005514	0.998725	0.001275	2:40:18
9	0.000869	0.004230	0.998852	0.001148	2:34:35

**Table 10:** Results of DenseNet 121.

Epoch	Train Loss	Valid Loss	Accuracy	Error Rate	Time
0	0.347987	0.165432	0.945033	0.054967	2:11:49
0	0.073293	0.024991	0.992475	0.007525	2:28:01
1	0.073558	0.013193	0.995791	0.004209	2:25:48
2	0.043090	0.016201	0.995664	0.004336	2:22:41
3	0.022192	0.009309	0.997449	0.002551	2:17:25
4	0.010602	0.002526	0.999235	0.000638	2:15:31
5	0.010123	0.011439	0.997449	0.002551	2:12:21
6	0.006923	0.001362	0.999362	0.000638	2:11:33
7	0.000690	0.000671	0.999745	0.000255	2:13:32
8	0.000207	0.000368	0.999745	0.000255	0:22:54
9	0.000408	0.000253	0.999872	0.000128	2:29:03

**Table 11:** Results of the DenseNet 169.

Epoch	Train Loss	Valid Loss	Accuracy	Error Rate	Time
0	0.288913	0.120378	0.960337	0.039663	2:44:14
0	0.059531	0.021940	0.992220	0.007780	3:00:51
1	0.057521	0.022707	0.993241	0.006759	2:55:58
2	0.058411	0.014491	0.995664	0.004336	2:52:39
3	0.031062	0.003245	0.999107	0.000893	2:51:29
4	0.015365	0.007066	0.998980	0.001020	2:51:46
5	0.004845	0.001774	0.999617	0.000383	2:51:51
6	0.000463	0.000892	0.999872	0.000128	2:57:59
7	0.000893	0.001066	0.999617	0.000383	3:27:21
8	0.000666	0.001539	0.999745	0.000255	3:50:44
9	0.000290	0.001327	0.999745	0.000255	3:25:05

**Table 12:** Results of the DenseNet 201.

Epoch	Train Loss	Valid Loss	Accuracy	Error Rate	Time
0	0.249325	0.106139	0.969902	0.030098	3:35:21
0	0.069267	0.013129	0.995791	0.004209	4:01:31
1	0.072991	0.021794	0.993623	0.006377	3:35:10
2	0.039306	0.014805	0.995536	0.004464	3:52:20
3	0.006408	0.006150	0.998215	0.001785	3:51:30
4	0.018931	0.004701	0.999235	0.000765	3:52:36
5	0.000636	0.003969	0.998597	0.000765	3:54:19
6	0.000636	0.003969	0.998597	0.001403	3:54:19
7	0.000484	0.111607	0.999235	0.000765	3:52:54
8	0.000013	0.001383	0.999490	0.000510	3:53:46
9	0.000450	0.001325	0.999490	0.000510	3:58:08

**Table 13:** Results of the AlexNet.

Epoch	Train Loss	Valid Loss	Accuracy	Error Rate	Time
0	1.442523	0.915624	0.705395	0.294605	14:18
0	0.365056	0.176912	0.947583	0.052417	11:48
1	0.186873	0.071719	0.980105	0.019895	13:04
2	0.109797	0.051216	0.982910	0.017090	13:26
3	0.087441	0.024694	0.992475	0.007525	14:14
4	0.061551	0.030640	0.990562	0.009438	12:47
5	0.031753	0.017091	0.994771	0.005229	13:32
6	0.008760	0.010013	0.997067	0.002933	13:27
7	0.003137	0.006157	0.998470	0.001530	13:04
8	0.001259	0.005411	0.998470	0.001530`	13:16
9	0.001717	0.004910	0.998725	0.001275	13:02

**Table 14:** Comparison between different systems.

Epoch	Train Loss	Valid Loss	Accuracy	Error Rate	Time
ResNet18	0.000053	0.001247	0.999490	0.000510	52:05
ResNet34	0.000018	0.000238	1.000000	0.000000	1:35:50
RedNet50	0.000536	0.000964	0.999745	0.000255	3:18:05
DenseNet 121	0.000408	0.000253	0.999872	0.000128	2:29:03
DenseNet 169	0.000290	0.001327	0.999745	0.000255	3:25:05
DenseNet 201	0.000450	0.001325	0.999490	0.000510	3:58:08
VGG16_bn	0.000009	0.000127	1.000000	0.000000	4:36:52
VGG19_bn	0.000004	0.000002	1.000000	0.000000	5:25:54
XresNet50	0.000869	0.004230	0.998852	0.001148	2:34:35
AlexNet	0.001717	0.004910	0.998725	0.001275	13:02
